# A novel immune-nutritional score predicts response to neoadjuvant immunochemotherapy after minimally invasive esophagectomy for esophageal squamous cell carcinoma

**DOI:** 10.3389/fimmu.2023.1217967

**Published:** 2023-10-25

**Authors:** Jifeng Feng, Liang Wang, Xun Yang, Qixun Chen, Xiangdong Cheng

**Affiliations:** ^1^ Department of Thoracic Oncological Surgery, Zhejiang Cancer Hospital, Hangzhou Institute of Medicine (HIM), Chinese Academy of Sciences, Hangzhou, China; ^2^ Zhejiang Provincial Research Center for Upper Gastrointestinal Tract Cancer, Zhejiang Cancer Hospital, Hangzhou, China; ^3^ Key Laboratory Diagnosis and Treatment Technology on Thoracic Oncology, Zhejiang Cancer Hospital, Hangzhou, China; ^4^ The Second Clinical Medical College, Zhejiang Chinese Medical University, Hangzhou, Zhejiang, China

**Keywords:** immune-nutritional index, esophageal squamous cell carcinoma, pathological complete response, neoadjuvant therapy, immunochemotherapy, prognosis

## Abstract

**Background:**

The role of neoadjuvant immunochemotherapy (NICT) has gradually attracted attention in recent years. To date, sensitive and reliable blood indicators to forecast the therapeutic response are still lacking. This study aimed to conduct a novel predictive score based on a variety of peripheral hematological immune-nutritional indicators to predict the therapeutic response in esophageal squamous cell carcinoma (ESCC) receiving NICT.

**Methods:**

There were 206 ESCC patients receiving NICT retrospectively recruited. With pathological complete response (pCR) as the dependent variable, independent risk variables of various peripheral blood immune-nutritional indexes were screened by logistic regression analyses to establish an integrative score.

**Results:**

By logical regression analyses, lymphocyte to monocyte ratio (LMR) and body mass index (BMI) were independent risk factors among all immune-nutritional indices. Then, an integrative score named BMI-LMR score (BLS) was established. Compared with BMI or LMR, BLS was related to complications, especially for respiratory complication (P=0.012) and vocal cord paralysis (P=0.021). Among all patients, 61 patients (29.6%) achieved pCR after NICT. BLS was significantly related to pCR [odds ratio (OR)=0.269, P<0.001)]. Patients in high BLS cohort demonstrated higher 3-year overall survival (OS) (89.9% vs. 67.9%, P=0.001) and disease-free survival (DFS) (81.2% vs. 62.1%, P=0.001). BLS served as an independent factor of DFS [hazard ratio (HR) =2.044, P =0.020) and OS (HR =2.960, P =0.019).

**Conclusion:**

The BLS, based on immune-nutritional indicators of BMI and LMR, employed as a straightforward, accurate, and useful indicator of pCR and prognostic prediction in ESCC patients undergoing NICT.

## Introduction

Esophageal cancer (EC) is one of the most common and aggressive cancers worldwide, with a high mortality and morbidity (1). The primary method of treatment for those with EC is still surgery, although the outcome is still subpar because of the early recurrence after treatment (2). Recently, with the continuous advancement of neoadjuvant therapy (NAT) in patients with EC, the therapy presents a combination of multiple therapeutic modalities, such as neoadjuvant chemotherapy (NCT) or neoadjuvant chemoradiotherapy (NCRT) (3, 4). However, the therapeutic outcomes for NAT remain unsatisfactory, mainly due to a high rate of recurrence and complication morbidity (5). Therefore, it is crucial to investigate more and more reliable NATs in EC. Having become a hotspot recently, immunotherapy, represented by several immune checkpoint inhibitors (ICIs), such as anti-PD-1/PD-L1 antibodies, significantly influenced the treatment strategies for advanced EC, especially in several well-known clinical studies (6, 7). Recently, more and more attention has been paid to the role of neoadjuvant immunochemotherapy (NICT). There is also a growing body of evidence that NICT is safe and effective in locally advanced EC (8–10).

Studies have shown that the pathological complete response (pCR) after NAT is a good predictor in evaluating the long-term survival in a variety of cancers (11, 12). A growing body of evidence also confirms that patients who achieve pCR could truly benefit from NAT. Otherwise, for patients with non-pCR, the prognosis may be worse than those with surgery alone due to the postoperative complications and the toxicity of NAT (13). Therefore, it is crucial to choose the best individualized treatment and to identify patients who can achieve pCR before NAT. Researchers have proposed several methods for predicting pCR in cancers, such as radiomics and genomics (14–16). However, due to the expensive and complex nature, these above methods are not widely used in daily clinical practice. Therefore, to find more economical and effective indices for pCR prediction before NAT is of great significance.

Several studies reported that cancer prognosis is associated not only with cancer behavior, but also with the immune and nutritional status (17, 18). Recently, a variety of immune and nutritional indexes, such as body mass index (BMI), neutrophil (NEU) to lymphocyte (LYM) ratio (NLR), LYM to monocyte (MON) ratio (LMR) and platelet (PLT) to LYM ratio (PLR), are closely related to the treatment response in various cancers (19–21). Moreover, systemic inflammation response index (SIRI), systemic immune-inflammation index (SII), prognostic nutritional index (PNI) and other hematological indexes were also applied in cancers for pCR prediction (22–24). However, the immune and nutritional status may be influenced by a variety of other conditions, which may lead to the biased results. Moreover, the above studies only evaluated one or two indices, and the predictive and prognostic values of these indicators for EC alone or in combination remain uncertain and need further validation.

To date, sensitive and reliable indicators to forecast the therapeutic response are still lacking. Furthermore, with the growing use of NICT in the EC treatment, therapeutic response and clinical results have received more attention. We hypothesized that a comprehensive index might be more valuable, reducing the potential bias and predicting the therapeutic response and prognosis. For patients with esophageal squamous cell carcinoma (ESCC), thus, the aim of this research was to investigate the impact of the combination of these indicators.

## Methods

### Patient selection

**Figure 1:**
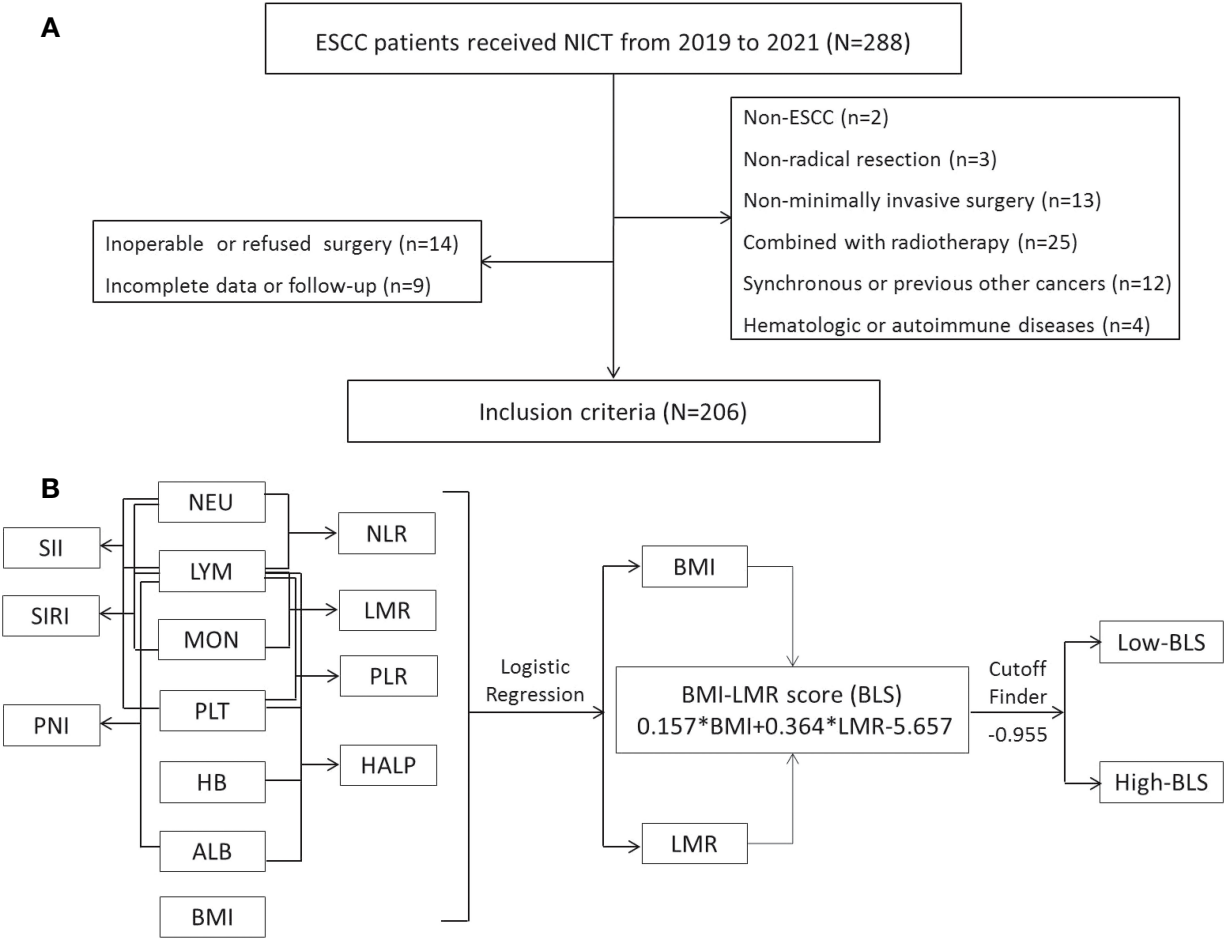
The flow diagram of selection of eligible patients **(A)**. Process diagram for BLS construction and risk stratification **(B)**.

ESCC patients who underwent NICT before radical minimally invasive esophagectomy (MIE) were recruited from 18 June 2019 to 28 December 2021. ESCC patients (cTNM II-IVA stages) between the ages of 18 and 75 who received NICT followed by radical MIE met the current study’s eligibility requirements. Patients were disqualified if they had diagnosed with non-ESCC, had other autoimmune or hematologic diseases, had other types of cancer, had received prior anticancer treatments, or had obtained a non-MIE or non-radical resection. **Figure 1A** displayed the selection flow. The Clavien-Dindo classification was used to refer to complications (25). The absence of cancer cells in both resected specimen and lymph nodes (LNs) was defined as pathological complete response (pCR) (26). Based on the 8th TNM edition, postoperative (ypTNM) and pretreatment (cTNM) staging was carried out (27).

### Therapeutic methods and follow-up

Prior to radical surgery, all the patients had two regimens of NICT every 3 weeks. The chemotherapeutic protocol included albumin paclitaxel (120mg/m^2^ on days 1 and 8) and carboplatin (area under the curve = 5mg/ml/min on day 1). The immunotherapy regimen, such as nivolumab at a dose of 3 mg/Kg, pembrolizumab at a dose of 2 mg/Kg, camrelizumab tislelizumab, or sintilimab at a dose of 200 mg, was delivered on day 1. A reassessment was conducted after NICT in order to evaluate patients who had any surgical contraindications. After the conclusion of the final NICT regimen, MIE with McKeown or Ivor Lewis was often scheduled to occur within 4-6 weeks (28). To date, there hasn’t been agreement on adjuvant therapy following NICT. Additionally, adjuvant immunotherapy given after surgery could dramatically improve the prognosis for patients who have undergone NCRT (29). The last follow-up was finished in December 2022, starting after the end of treatment. Patients were checked on at regular intervals (after the first two years, every three months; after three to five years, every six months; and after five years, every year).

### Immune-nutritional status assessment

The pretreatment peripheral blood indexes, such as hemoglobin (HB), albumin (ALB), NEU, PLT, LYM, and MON, were obtained from our electronic medical database. One week before to NICT, all the peripheral blood indexes were collected. The LMR, PLR, and NLR were defined as LYMs divided by MONs, PLTs divided by LYMs, and NEUs divided by LYMs, respectively (19–21). The SIRI, PNI, and SII were calculated by the following formula: PNI= ALB (g/L) +5 × LYM (10^9^/L), SII= PLT × NEU/LYM and SIRI= MON × NEU/LYM (22–24). According to previous published study, the HALP was defined as: HALP=HB×ALB×LYM/PLT (30). Sarcopenia, which has a detrimental effect on life quality and raises the risk of death, is characterized by a progressive, systematic decline in skeletal muscle strength and mass (31). The skeletal muscle index (SMI, cm^2^/m^2^), defined as the total muscle area shown on a computed tomography (CT) at the level of the third lumbar vertebra divided by the height squared, is a regularly used method to evaluate sarcopenia (31). The SMI ≤ 52.4cm^2^/m^2^ for males and ≤38.5 cm^2^/m^2^ for females was used to define sarcopenia (32).

### Statistical analysis

R 4.1.2 software and SPSS 20.0 was used to conduct statistical analysis and P values <0.05 represented statistically significant. Using PCR as the independent variable, univariate logistic regression analysis was used to select variables. To investigate the multicollinearity among various independent variables, variance inflation factor (VIF) and tolerance measures were used (33). A VIF > 5 or tolerance <0.1 indicated severe collinearity of the variables (34). Then, all variables with P < 0.1 from univariate logistic analysis were analyzed in multivariate analysis. Independent risk variables from peripheral blood indicators in multivariate logical regression analyses were performed to construct an integrative score. The predictive accuracy of the integrative score and other peripheral blood indicators were compared based on the receiver operator characteristic (ROC) curves. The areas under the curves (AUCs) between BLS and other traditional indices were compared with Delong’s test. Taking pCR as a dependent variable in the study, the cutoff finder (35) software, a bundle of optimization and visualization methods for cutoff determination, was performed to measure the thresholds. The predictors of pCR prediction were evaluated by logistic analyses. In order to determine the various predictive parameters for overall survival (OS) and disease-free survival (DFS), Cox regression analyses were also performed.

## Results

### Patient characteristics

The baseline characteristics and various hematological indices are listed in **Table S1**. A total of 206 patients with ESCC who underwent NICT followed by MIE were recruited in the current study. The patient cohort included 16 (7.8%) females and 190 (92.2%) males. The mean age was 63.2 ± 6.7 years (range: 47-75 years). Among the 206 cases, there were 20 (9.7%) cases, 121 (58.7%) cases and 65 (31.6%) cases located in the upper, middle and lower third of the esophagus, respectively. Among all patients, 32 (15.5%) cases had well differentiation, 92 (44.7%) cases had moderated differentiation and 82 (39.8%) cases had poor differentiation, respectively. Ninety-five patients were classified as having sarcopenia (46.1%). Patients in the study who were classified as having cTNM stage III (67.0%, n=138) were the most prevalent forms. There were 11 (5.3%), 29 (14.1%), 113 (54.9%), 14 (6.8%) and 39 (18.9%) patients who treated with nivolumab, pembrolizumab, camrelizumab, sintilimab and tislelizumab, respectively. The mean number of total, positive and negative LNs were 22.1 ± 8.9 (range: 8-57), 1.17 ± 2.19 (range: 0-12) and 21.0 ± 8.8 (range: 6-53), respectively. Adjuvant treatments were treated in 91 (44.2%) cases, including 67 (32.5%) cases in immunotherapy and 24 (11.7%) cases in chemoradiotherapy. The median follow-up time was 17 months (range: 7-38 months). Fifty-five (26.7%) patients relapsed and 31 (15.0%) cases died in the current study.

### Construction of BLS by logical regression

**Figure 2:**
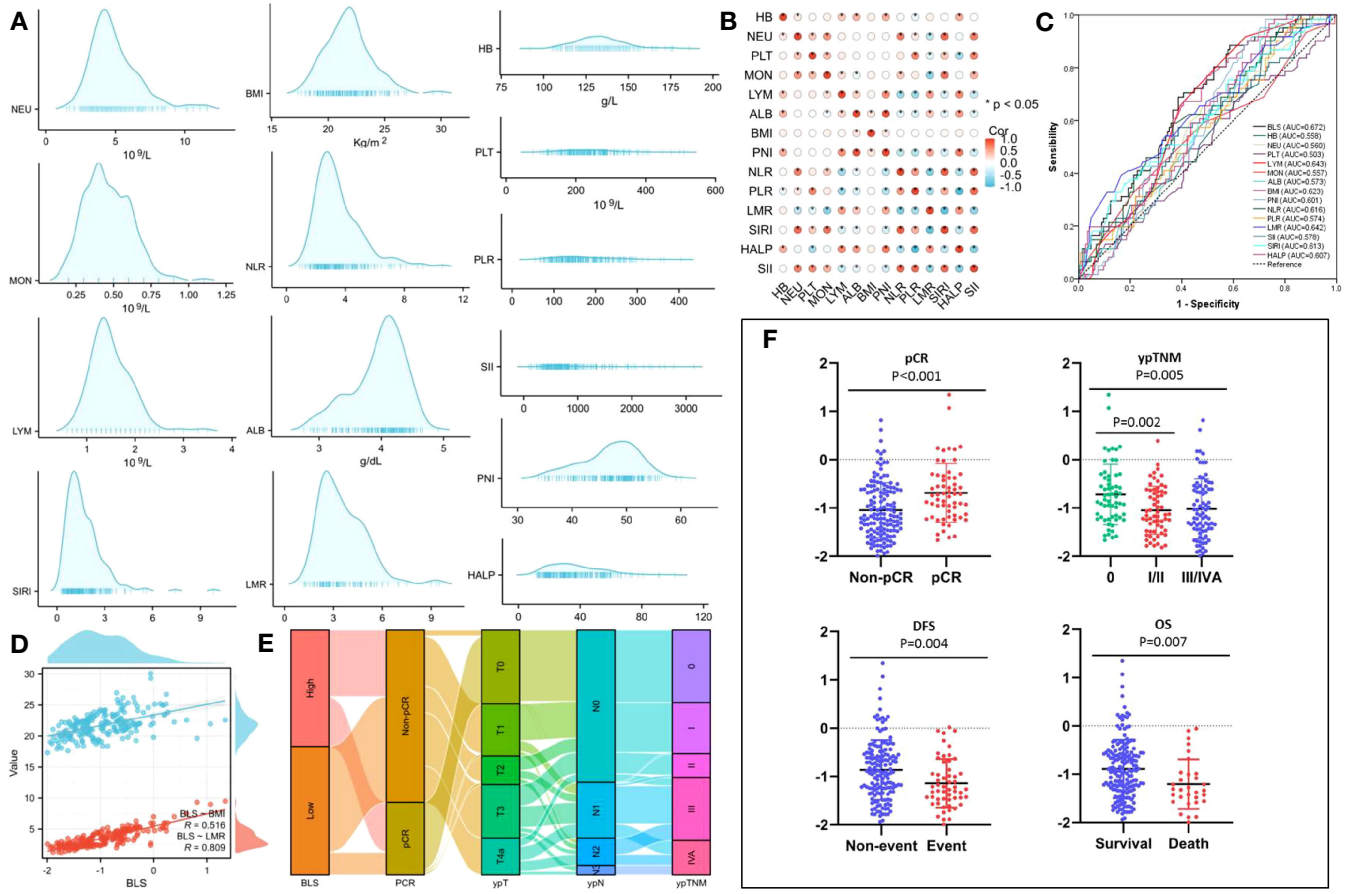
The distribution of all 14 immune-nutritional variables **(A)**. The heatmap correlations of all indexes **(B)**. The AUCs of all indexes **(C)**. The correlations between BLS, BMI and LMR **(D)**. The sankey diagram of BLS **(E)**. The scatter diagram of BLS grouped by pCR, ypTNM, DFS and OS **(F)**.

The process diagram of BLS is shown in **Figure 1B**. Firstly, univariate logistic analysis was used to select possible variables. Secondly, the tolerance and VIF was introduced through stepwise variable selection to address the collinearity. The results revealed that there was no obvious collinearity between the variables (VIFs between 1.001 and 1.803; tolerances between 0.553 and 0.999) (**Table S2**). Finally, variables with P value less than 0.1 from univariate logistic analysis were then analyzed in multivariate analysis. Based on the analyses, BMI and LMR, as continuous variables, were significant independent predictors (**Table S3**). Subsequently, an integrative score based on BMI and LMR, named BMI-LMR score (BLS), was established with the logistic regression equation as follows: Y_BLS_ = 0.157 × BMI + 0.364 × LMR - 5.657 based on the logistic regression equation. The distribution of all immune-nutritional variables is shown in **Figure 2A**. The correlation diagrams between BLS and other hematological indices are shown in **Figure 2B**. The area under the curve (AUC) comparisons between BLS and other conventional immune-nutritional scores are analyzed in **Figure 2C**. The AUCs between BLS and other traditional immune-nutritional indices were compared by Delong’s test (**Table S4**). Although BLS had the largest AUC (0.672 in continuous) compared with its component of BMI and LMR, the results suggested that BLS was not superior to LMR (P=0.204) or BMI (P=0.283) in terms of the discrimination performance for predicting pCR. It’s worth noting that compared with some traditional indicators (such as PLR, SII and SIRI), BLS had obvious advantages in indicating pCR predictive ability. Compared with PLR, SII and SIRI, however, BMI or LMR didn’t demonstrate the discrimination performance for predicting pCR. Thus, BLS may be used as a complement to BMI or LMR in predicting PCR. Positive correlations were found between BLS and BMI and LMR, respectively (**Figure 2D**). The sankey diagram was performed to analyze the associations between BLS and pCR and tumor stage (**Figure 2E**). A scatter diagram revealed that patients in low-BLS group were related to a significantly lower pCR rate, higher tumor burden and higher risk of death (**Figure 2F**).

### Complications and characteristics grouped by BLS

Based on the **Figure S1**, the optimal cut-off value for BLS was -0.955 with the odds ratio (OR) =2.72 and 95% confidence interval (CI) =1.60-4.63 (P<0.001). The specificity and sensitivity for the cut-off value was 61.4% and 68.9%, respectively. All patients then were split into two cohorts: High BLS which included 98 cases (47.6%) and Low BLS which included 108 cases (52.4%). **Table 1** showed the clinical characteristics grouped by BLS. Patients in low-BLS group had longer tumor length than that in high-BLS group (2.09 ± 2.02 cm vs. 1.42 ± 1.80 cm, P=0.012). Similarly, patients with low-BLS tended to have more advanced ypTNM, ypN, and ypT stages, respectively (P=0.008, P=0.035, and P<0.001, respectively). As expected, the BLS was likewise significantly correlated with both LMR and BMI (P<0.001 for each). In addition, BLS was also significantly related to sarcopenia (P=0.022). The intraoperative characteristics grouped by BMI, LMR and BLS were shown in **Figures S2A–C**. Patients with low-BLS had longer operation time (227.1 ± 31.3 min vs. 216.1 ± 39.3 min, P=0.027) and hospital stay after surgery (15.2 ± 8.8 day vs. 12.8 ± 4.9-day, P=0.019), respectively. However, no significant differences regarding total harvested LNs, positive LNs and negative LNs were found between the groups. Regarding to the major postoperative complications (**Figure S2D–F**), patients in the low-BLS group had higher incidences of vocal cord paralysis (14.8% vs. 5.1%, P=0.021) and respiratory complications (28.7% vs. 14.3%, P=0.012). Except operation time, no significant differences were found between the two groups based on BMI or LMR, suggesting BLS may be closely related to postoperative complications.

**Table 1:** Patient characteristics grouped by BLS in ESCC.

	Low-BLS (n=108)	High-BLS (n=98)	P-value
Sex (female/male, n, %) Age (mean ± SD, years) Hypertension (yes/no, n, %) Diabetes (yes/no, n, %) Smoking history (yes/no, n, %) Drinking history (yes/no, n, %) Tumor location (n, %) Upper Middle Lower Differentiation Well Moderate Poor Vessel invasion (yes/no, n, %) Perineural invasion (yes/no, n, %) Tumor length (mean ± SD, cm) Sarcopenia (yes/no, n, %) cTNM stage (n, %) II III IVA ypT stage (n, %) T0 T1 T2 T3 T4a ypN stage (n, %) N0 N1 N2 N3	4 (3.7)/104 (96.3) 63.2 ± 7.1 27 (25.0)/81 (75.0) 6 (5.6)/102 (94.4) 80 (74.1)/28 (25.9) 83 (76.9)/25 (23.1) 10 (9.3) 63 (58.3) 35 (32.4) 16 (14.8) 50 (46.3) 42 (38.9) 13 (12.0)/95 (88.0) 22 (20.4)/86 (79.6) 2.09 ± 2.02 58 (53.7)/50 (46.3) 14 (13.0) 76 (70.4) 18 (16.6) 22 (20.4) 19 (17.6) 16 (14.8) 34 (31.5) 17 (15.7) 61 (56.5) 26 (24.1) 15 (13.9) 6 (5.5)	12 (12.2)/86 (87.8) 63.2 ± 6.2 31 (31.6)/67 (68.4) 4 (4.1)/94 (95.9) 66 (67.3)/32 (32.7) 64 (65.3)/34 (34.7) 10 (10.2) 58 (59.2) 30 (30.6) 16 (16.3) 42 (42.9) 40 (40.8) 10 (10.2)/88 (89.8) 13 (13.3)/85 (86.7) 1.42 ± 1.80 37 (37.8)/61 (62.2) 21 (21.4) 62 (63.3) 15 (15.3) 40 (40.8) 25 (25.5) 8 (8.2) 11 (11.2) 14 (14.3) 67 (68.4) 21 (21.4) 8 (8.2) 2 (2.0)	0.022 0.965 0.290 0.867 0.289 0.067 0.948 0.878 0.677 0.175 0.012 0.022 0.271 <0.001 0.035
ypTNM stage (n, %) 0 I II III IVA Immunotherapy (n, %) Camrelizumab Pembrolizumab Nivolumab Sintilimab Tislelizumab Adjuvant treatment (n, %) None Immunotherapy Chemoradiotherapy Total lymph nodes (n, %) Positive lymph nodes (n, %) Negative lymph nodes (n, %) BMI (mean ± SD, Kg/m^2^) LMR (mean ± SD)	21 (19.4) 23 (21.3) 15 (9.3) 31 (28.7) 18 (16.7) 61 (56.5) 16 (14.8) 7 (6.5) 7 (6.5) 17 (15.7) 63 (58.3) 32 (29.6) 13 (12.1) 21.8 ± 8.4 1.41 ± 2.39 20.4 ± 8.4 20.93 ± 1.56 2.67 ± 0.73	40 (40.8) 20 (20.4) 5 (5.2) 22 (22.4) 11 (11.2) 52 (53.1) 13 (13.3) 4 (4.1) 7 (7.1) 22 (22.4) 52 (53.1) 35 (35.7) 11 (11.2) 22.5 ± 9.6 0.91 ± 1.92 21.6 ± 9.2 22.76 ± 2.08 4.53 ± 1.38	0.008 0.735 0.647 0.549 0.102 0.310 <0.001 <0.001

BLS, BMI-LMR score; ESCC, esophageal squamous cell carcinoma; SD, standard deviation; TNM, tumor node metastasis; BMI, body mass index; LMR, lymphocyte to monocyte ratio.

### Logistic analyses for pCR prediction

Based on postoperative histology, pCR was attained in 61 patients (29.6%) following NICT. Patients in high-BLS group had higher pCR rate than those in low-BLS group (42.9% vs. 17.6%, P<0.001). Similarly, patients with pCR had higher levels of BLS than that in non-pCR group (-0.688 ± 0.609 vs. -1.045 ± 0.568, P<0.001). The results of logistic regression analyses are shown in **Table S5**. Pretreatment BLS served as a sensitive and effective predictive index for pCR prediction (OR=0.269, 95% CI=0.138-0.527, P<0.001). Although sarcopenia was not an independent predictive index of pCR in those with ESCC receiving NICT, it suggested a trend in this regard (P=0.062; **Table S5**). Additionally, differentiation (P=0.029) and cTNM (P=0.040) were also shown to be important predictors of pCR prediction.

### Cox analyses for DFS and OS prediction

**Figure 3:**
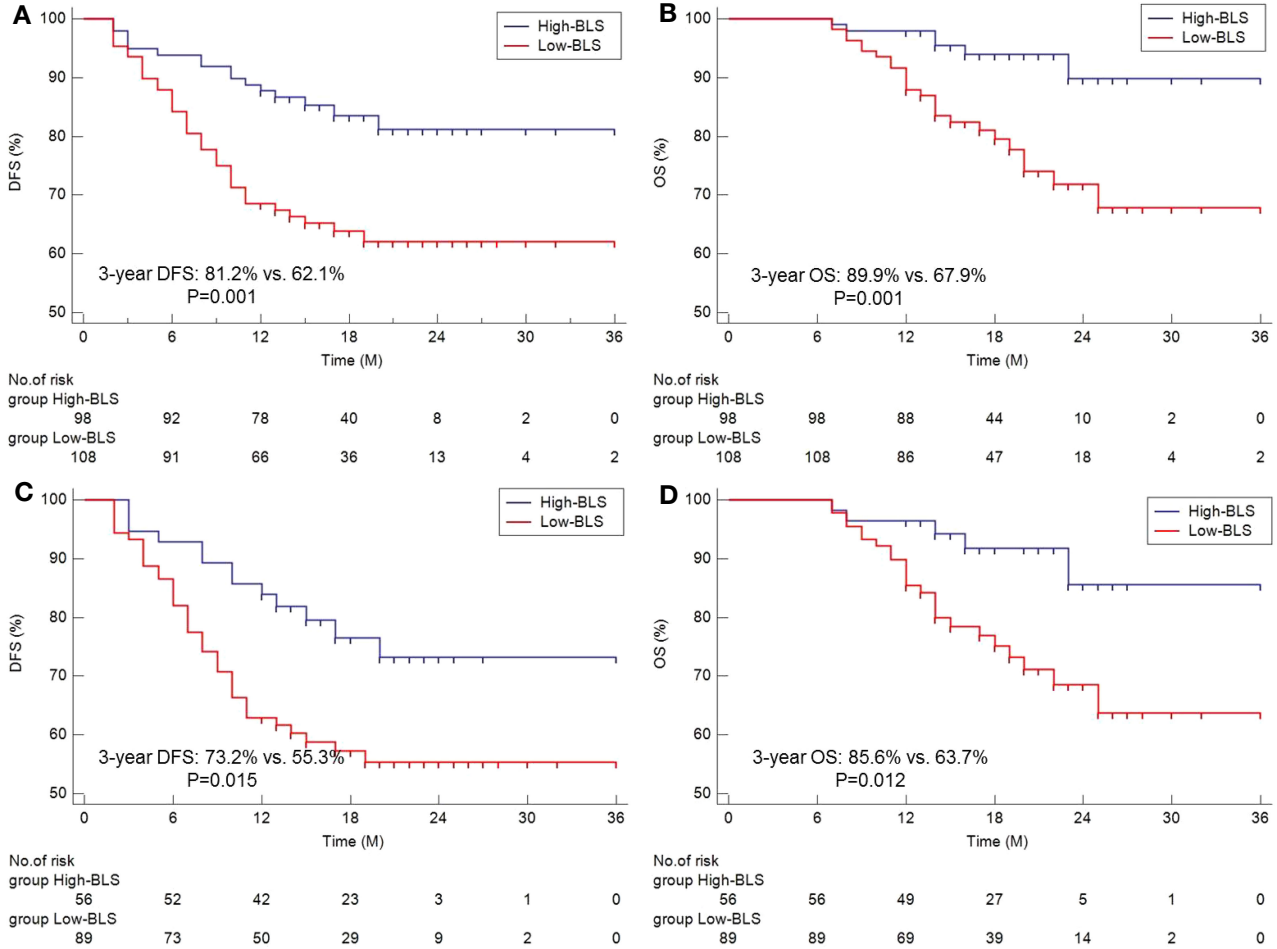
The Kaplan-Meier curves of DFS **(A)** and OS **(B)** grouped by BLS. Subgroup analysis of Kaplan-Meier curves of DFS **(C)** and OS **(D)** in non-pCR patients.

The survival curves of DFS and OS grouped by BLS are shown in **Figures 3A, B**. Patients in high BLS group had higher 3-year OS (89.9% vs. 67.9%, P=0.001) and 3-year DFS (81.2% vs. 62.1%, P=0.001) than those with low BLS, respectively. For patients with non-pCR, subgroup analysis also revealed the significant results (**Figures 3C, D**). In this study, no significant differences were found between the different immunotherapy regimen groups (**Figures S3A, B**). On terms of postoperative adjuvant treatment, moreover, there was no statistical difference between the different treatments (**Figures S3C, D**). However, subgroup analysis revealed the statistical differences in postoperative adjuvant therapy in those with non-PCR and LN-positive patients, especially in DFS (**Figures S3E–H**). The Cox results are shown in **Tables S6**, **S7**. In the present study, tumor location, vessel invasion, ypN stage, sarcopenia, and BLS were significantly associated with DFS while sarcopenia, ypN stage, and BLS were significantly related to OS, respectively. According to the Cox analyses results, the BLS was an independent index for DFS [hazard ratio (HR) =2.044, 95% CI =1.120-3.731, P =0.020)] and OS (HR =2.960, 95% CI =1.198-7.315, P =0.019). However, immunotherapy regimen or adjuvant treatment was not an independent factor in DFS or OS (**Tables S6**, **S7**).

## Discussion

NICT is being increasingly used to treat cancer patients to reduce the tumor burden, reduce the tumor size and increase the likelihood of radical resection (10). Previous published studies have also confirmed that NICT can increase pCR rate, improve surgical resection rate and control drug toxicity in EC (8–10). However, it is currently difficult to predict the therapeutic response to NICT in EC due to the absence of widely available sensitive and efficient biomarkers. In ESCC patients receiving NICT, the current study examined the immune-nutritional status of BLS based on BMI and LMR. Additionally assessed were the connections between the BLS, prognosis and treatment effect. The outcomes reported that BLS outperformed a number of blood indices in terms of its ability to predict pCR. The outcomes also revealed a strong correlation between BLS, a sensitive and reliable predictor of prognosis, and postoperative complications.

Poor nutrition is highly implicated in the cancer pathogenesis and affects the therapeutic tolerance, quality of life and prognosis (36). Previous study indicated that adipose cells are involved in the development and maintenance of inflammation by secreting a variety of chemokines and cytokines (37). Furthermore, studies have also shown that obesity, on the one hand, increases T-cell aging, leading to higher PD-1 expression. On the other hand, the tumor responsive in obesity may be significantly improved due to the PD-1-mediated T-cell dysfunction (38). As a major substitute for nutritional status, historically, the correlations between BMI and prognosis in EC remain contradictory (39–41). A lower BMI not only increased postoperative complications but also impaired OS and DFS in ESCC after surgery (39). However, another study revealed that a high BMI was not related to the increased overall morbidity following surgery. Although a better survival was found in patients with high BMI, the authors suggested that this might be due to their relatively lower pathological staging (40). Moreover, another study including elderly ESCC patients with MIE, the authors also demonstrated that there was insufficient evidence to demonstrate the associations between BMI and perioperative and oncological adverse outcomes (41). Furthermore, a meta-analysis reported that no significant differences in mortality were observed among patients in different BMI (42). In addition, for patients with ICIs treatment, a meta-analysis revealed that pretreatment BMI was remarkably correlated with the survival (43). For EC patients receiving NICT, however, there is still no consensus about the impact of BMI.

The immunological status of peripheral blood in cancer patients remains unclear, just as its role as a predictive/prognostic index. LMR, as an immune index of LYMs to MONs ratio, reflects the interaction taking place between immune cells and cancer cells. The mechanism of LYMs destroying and affecting the metastasis and proliferation of tumor cells is through the expression of tumor-infiltrating LYMs after infiltrating into the tumor microenvironment (44). Furthermore, an immune response to tumor cells is established when LYMs are involved in immune regulation in the tumor microenvironment (45). Tumor-associated macrophages derived from MONs can promote tumor angiogenesis, aggression and metastasis (46, 47). The relationship between LMR and cancer development, progression and metastasis has already been proved in a variety of cancers (48, 49). For patients with EC, the prognostic value of LMR was confirmed recently in a meta-analysis, indicating that low LMR was associated with advanced characteristics and poor prognosis ESCC (50). Moreover, LMR was also confirmed as a predictor in EC receiving NAT (20, 51, 52). Pretreatment LMR may predict pCR after NCRT in a study of 87 ESCC patients (51). In addition LMR was confirmed as a predictor in ESCC receiving NICT. The results indicated that LMR was significantly related to pCR after NICT in several cancers (20, 52).

Due to variations in therapeutic intervention tolerance, immune and nutritional status is critical for cancer prognosis and treatment (17, 18). Identifying predictive indices before treatment to help determine the optimal time of therapy and surgery is important. The majority of clinicians evaluated the immunological or nutritional status solely based on single parameter, which produced conflicting results. In order to reflect the overall condition of immune-nutritional status, an integrated index should be established. In this investigation, the BLS, which reflected a number of characteristics of immune-nutritional status, was made up of two commonly used clinical indices (BMI and LMR). In this investigation, it was beneficial for doctors to give EC patients more specific information about their overall condition of immune-nutrition. This significantly decreased postoperative complications, improved therapeutic response and prognosis. However, the further results of nutritional intervention in our study need to be confirmed in the future.

Low-BLS patients had higher incidences of respiratory complications and vocal cord paralysis in the current study. These morbidities represent the most common complications after EC surgery, which seriously affected the recovery and prognosis (53). Several risk factors for respiratory morbidity have been confirmed, such as cardiovascular comorbidities, smoking habits and longer hospital stay (54). Surgical injuries and stresses occurring in the region near the recurrent laryngeal nerve (RLN) were most risk factors of vocal cord paralysis in EC (55). Patients in the current study with vocal cord paralysis were injured by surgery. Therefore, the intrinsic relationship between BLS and vocal cord paralysis may be based on the following reasons. Firstly, patients in low BMI are prone to suffer complications after surgery due to poor nutrition status (56). Secondly, studies have been reported that several nutritional and inflammatory indices based on LYMs were associated with high incidence of postoperative complications (57, 58). Thirdly, as described in our study, BLS was correlated with tumor stage. Patients in lower BLS tend to have higher tumor stage and mediastinal LN metastasis (LNM), especially for those with LNM beside RLN. Therefore, the probability of vocal cord paralysis in these patients during surgical dissection will be greatly increased. In addition, BLS was also significantly related to sarcopenia in the current study. According to a theory put forth by researchers, sarcopenia may be the result of prolonged catabolism or increased metabolic activity caused by a more aggressive tumor biology, which would then result in systemic inflammation, muscular atrophy, and a worse prognosis (59). Sarcopenia was also found to be an independent predictor of DFS (P=0.020) and OS (P=0.019) in those with NICT in ESCC, according to this study, which was in line with a recently published meta-analysis (60). According to this meta-analysis, sarcopenia was present in 48.1% of EC patients and was linked to worse OS and DFS.

In the study, the results reported that BLS was closely associated with pCR, although BLS was not superior to its component of LMR or BMI in terms of the discrimination performance for predicting pCR. However, some advantages should be noticed. Firstly, BLS had some obvious advantages in indicating pCR predictive ability compared with some traditional indicators (such as PLR, SII and SIRI). Secondly, BLS was also more significant in predicting postoperative complications than BMI and LMR. Thirdly, BLS was an effective and sensitive score of OS and DFS. It’s worth noting that the AUCs comparisons didn’t show difference between BLS and BMI or LMR, possibly due to the small sample size, the BLS derived from BMI and LMR, and the partial coincidence of CIs. Therefore, most prospective studies with large samples are needed to validate the BLS results.

This research has some limitations. First, due to the relatively limited sample size and the single-center retrospective observational method, selection bias was unavoidable. Second, the discrepancy may have distinct effects due to the various immunotherapy regimens. Third, although this study adopted strict inclusion and exclusion criteria, hematological indexes may be affected by other conditions. Fourth, although there was a substantial relationship between BLS and prognosis, results from long-term follow-up need to be validated. Fifth, the mechanisms underlying BLS’s biological behavior in NICT are still not completely understood. Moreover, the current study only analyzed the impact of pretreatment hematological indexes in ESCC, but lacking the effect of these relevant indices during or after NICT on prognosis. In addition, although this manuscript showed that the prognosis is related to BLS, it couldn’t be ruled out that the prognosis of patients is related to the tumor itself. Therefore, additional prospective studies are required to corroborate the actual results.

## Conclusions

In summary, pretreatment BLS may be employed as a straightforward, accurate, and useful indicator of pCR and prognostic prediction in ESCC patients undergoing NICT. Clinicians may be able to treat ESCC cancer patients more individually with the help of BLS stratification. However, more information about the mechanism between BLS and prognosis of EC needs to be further explored.

## Data availability statement

The original contributions presented in the study are included in the article/**Supplementary Material**. Further inquiries can be directed to the corresponding authors.

## Ethics statement

The study was performed in accordance with the Helsinki Declaration and was approved by the ethics committee of Zhejiang Cancer Hospital (IRB2020-320). The studies were conducted in accordance with the local legislation and institutional requirements. The ethics committee/institutional review board waived the requirement of written informed consent for participation from the participants or the participants’ legal guardians/next of kin because Informed consent was waived because this was a retrospective study and all data were anonymous.

## Author contributions

QC and XC contributed and designed the study. JF and LW drafted the manuscript. JF, LW and XY contributed to data collect. LW and XY interpreted and analyzed the data. QC and XC reviewed the manuscript for important intellectual content critically. All authors contributed to the article and approved the submitted version.

## Supplementary material

Supplementary Figure 1 The optimum cut-off value based on the cutoff finder with the dependent variable of pCR for BLS **(A)**, BMI **(B)** and LMR **(C)**.

Supplementary Figure 2 The intraoperative characteristics grouped by BLS **(A)**, BMI **(B)** and LMR **(C)**. The major postoperative complications grouped by BMI **(D)**, LMR **(E)** and BLS **(F)**.

Supplementary Figure 3 Kaplan-Meier curves of DFS **(A)** and OS **(B)** grouped by immunotherapy regimen. Kaplan-Meier curves of DFS **(C)** and OS **(D)** grouped by adjuvant treatment. Subgroup analysis of Kaplan-Meier curves of DFS **(E)** and OS **(F)** in non-pCR patients. Subgroup analysis of Kaplan-Meier curves of DFS **(G)** and OS **(H)** in LN-positive patients.
