# Supplementary figures and images for: A novel immune-nutritional score predicts response to neoadjuvant immunochemotherapy after minimally invasive esophagectomy for esophageal squamous cell carcinoma
Source: Front Immunol. 2023 Oct 25;14:1217967. doi: 10.3389/fimmu.2023.1217967 (PMC10634314; doi:10.3389/fimmu.2023.1217967)

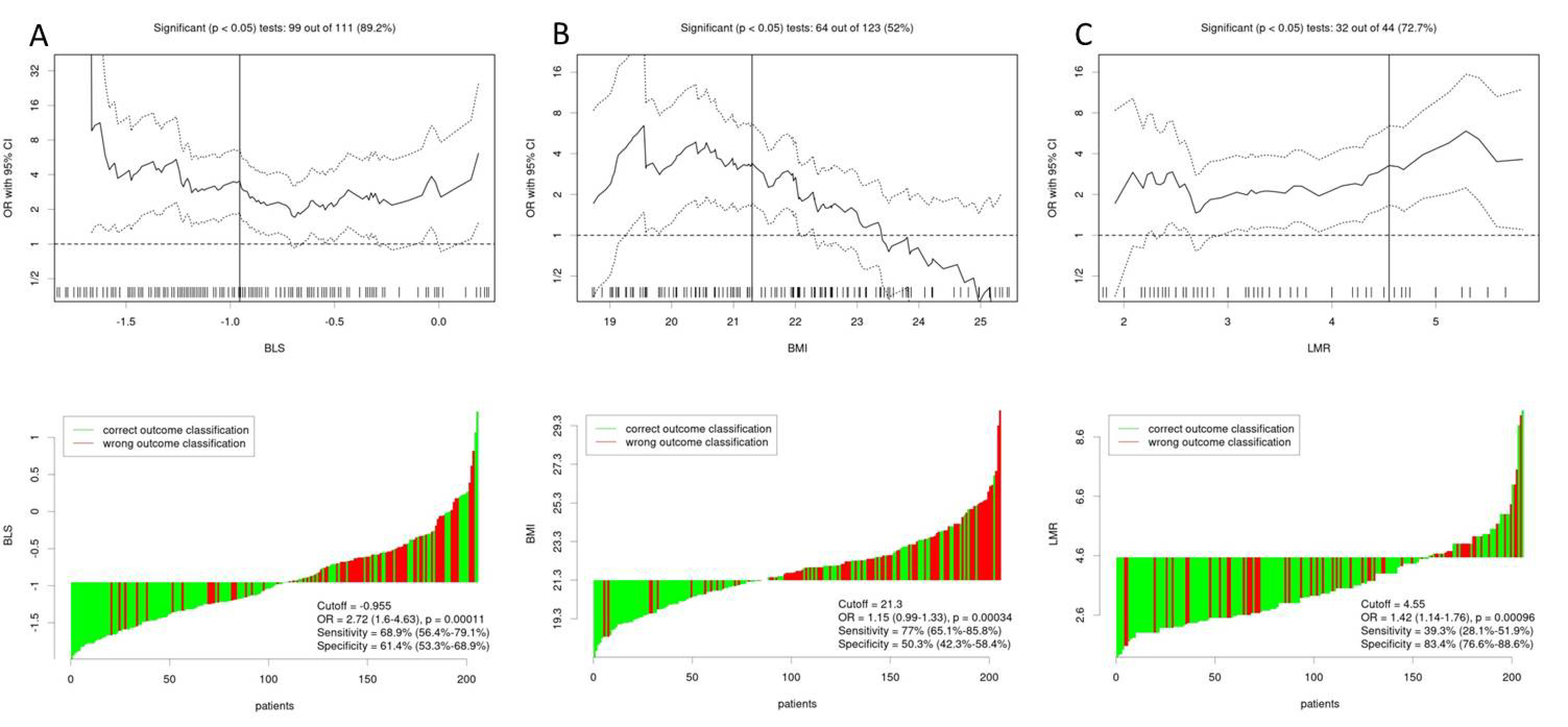

Supplement: Supplementary Figure 1 — The optimum cut-off value based on the cutoff finder with the dependent variable of pCR for BLS (A), BMI (B) and LMR (C). [file Image_1.jpeg]

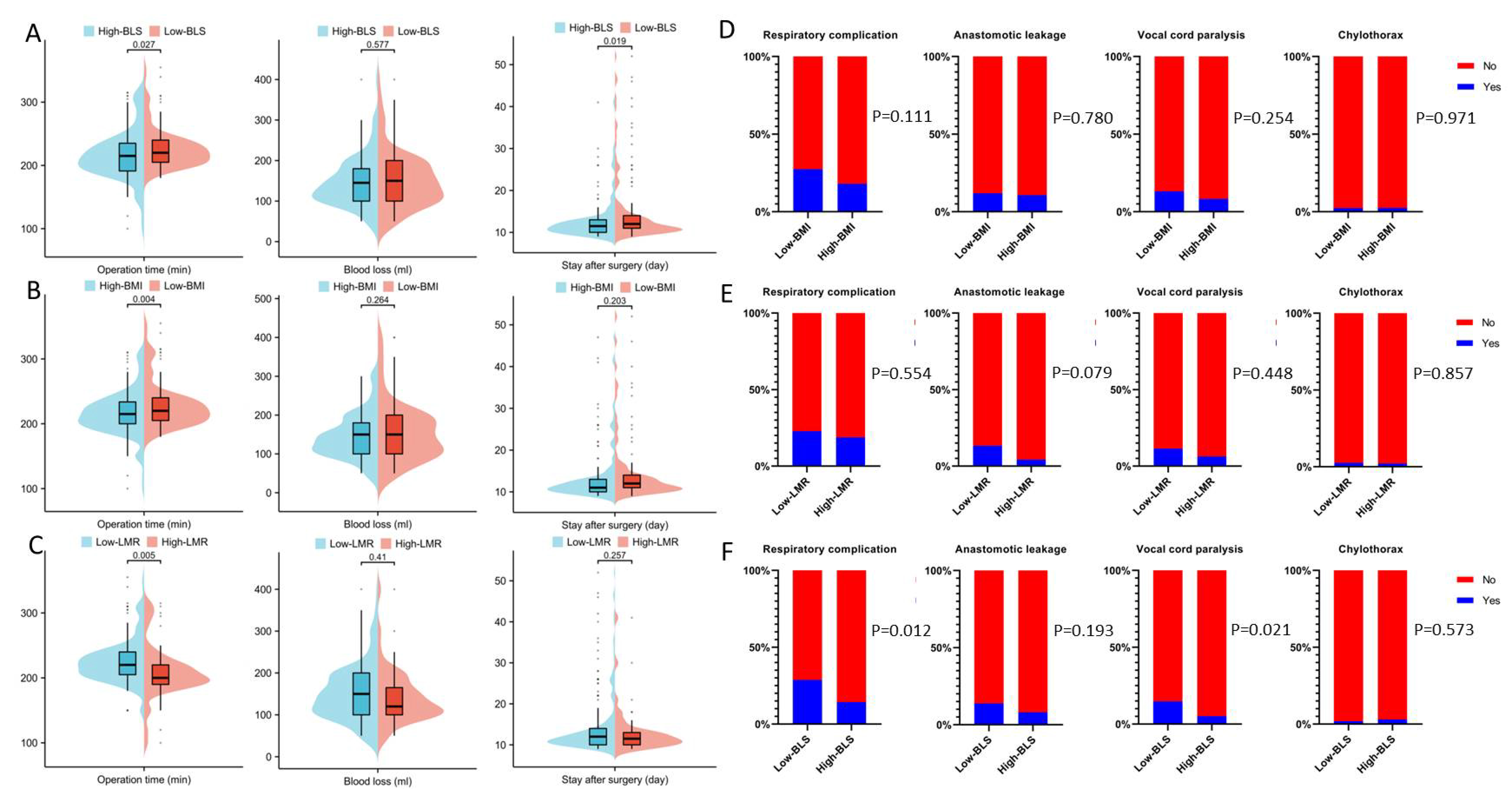

Supplement: Supplementary Figure 2 — The intraoperative characteristics grouped by BLS (A), BMI (B) and LMR (C). The major postoperative complications grouped by BMI (D), LMR (E) and BLS (F). [file Image_2.jpeg]

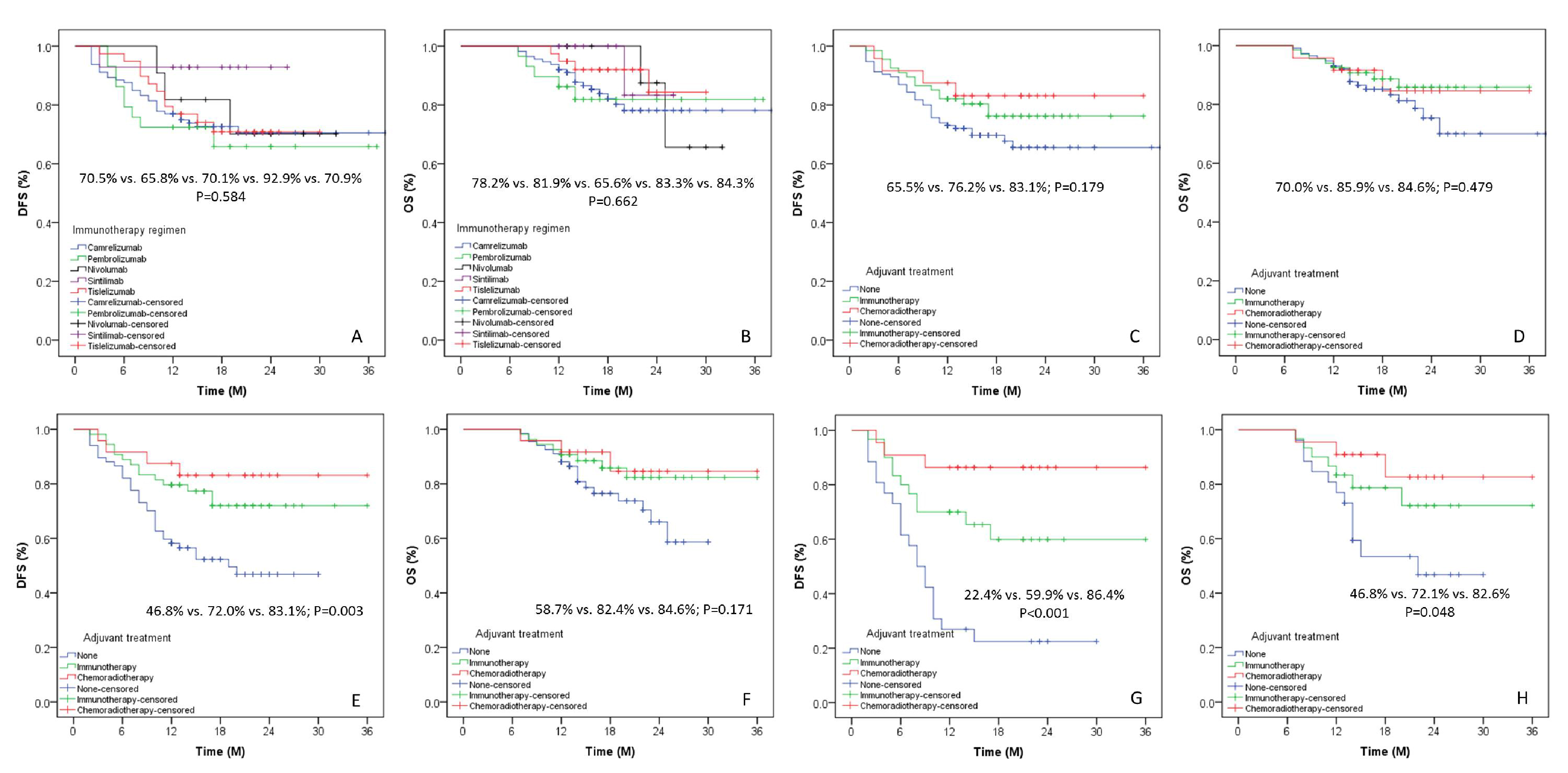

Supplement: Supplementary Figure 3 — Kaplan-Meier curves of DFS (A) and OS (B) grouped by immunotherapy regimen. Kaplan-Meier curves of DFS (C) and OS (D) grouped by adjuvant treatment. Subgroup analysis of Kaplan-Meier curves of DFS (E) and OS (F) in non-pCR patients. Subgroup analysis of Kaplan-Meier curves of DFS (G) and OS (H) in LN-positive patients. [file Image_3.jpeg]
