# Supplementary material for: A novel immune-nutritional score predicts response to neoadjuvant immunochemotherapy after minimally invasive esophagectomy for esophageal squamous cell carcinoma
Source: Front Immunol. 2023 Oct 25;14:1217967. doi: 10.3389/fimmu.2023.1217967 (PMC10634314; doi:10.3389/fimmu.2023.1217967)
Supplement: Supplementary file 5 [file Table_2.docx]

**Table S1** **Baseline clinical characteristics and hematological indices of ESCC**

|  | Total (n=206) |
| --- | --- |
| Sex (female/male, n, %)  Age (mean ± SD, years)  Hypertension (yes/no, n, %)  Diabetes (yes/no, n, %)  Smoking history (yes/no, n, %)  Drinking history (yes/no, n, %)  Tumor location (U/M/L, n, %)  Differentiation (W/M/P, n, %)  cTNM stage (II/III/IVa, n, %)  BMI (mean ± SD, range, Kg/m^2^)  Hemoglobin (mean ± SD, g/L)  Neutrophil (mean ± SD, 10^9^/L)  Platelet (mean ± SD, 10^9^/L)  Monocyte (mean ± SD, 10^9^/L)  Lymphocyte (mean ± SD, 10^9^/L)  Albumin (mean ± SD, g/dL)  PNI (mean ± SD)  NLR (mean ± SD) | 16 (7.8)/190 (92.2)  63.2 ± 6.7 (47-75)  58 (28.2)/148 (71.8)  10 (4.9)/196 (95.1)  146 (70.9)/60 (29.1)  147 (71.4)/59 (28.6)  20 (9.7)/121 (58.7)/65 (31.6)  32 (15.5)/92 (44.7)/82 (39.8)  35 (17.0)/138 (67.0)/33 (16.0)  21.8 ± 2.0 (17.3-30.1)  133.1 ± 14.6 (92-186)  4.94 ± 1.76 (1.5-11.6)  237.3 ± 73.3 (77-510)  0.46 ± 0.15 (0.2-1.1)  1.50 ± 0.44 (0.6-3.5)  3.95 ± 0.43 (2.75-4.92)  47.0 ± 5.3 (33.8-60.1)  3.51 ± 1.56 (0.88-10.60) |
| PLR (mean ± SD)  LMR (mean ± SD)  SII (mean ± SD)  SIRI (mean ± SD)  HALP (mean ± SD) | 167.8 ± 63.5 (55.5-405.0)  3.56 ± 1.43 (1.18-9.50)  851.7 ± 516.8 (99.7-3149.8)  1.70 ± 1.19 (0.26-9.82)  36.8 ± 16.9 (11.43-101.58) |

**Abbreviation:** ESCC: esophageal squamous cell carcinoma; SD: standard deviation; BMI: body mass index; U/M/L: upper/middle/lower; W/M/P: well/moderate/poor; TNM: tumor node metastasis; PNI: prognostic nutritional index; NLR: neutrophil to lymphocyte ratio; PLR: platelet to lymphocyte ratio; SII: systemic immune-inflammation index; LMR: lymphocyte to monocyte ratio; SIRI: systemic inflammation response index; HALP: hemoglobin albumin lymphocyte platelet.

**Table S2** **Univariate logistic analyses for pCR prediction based on various indices in ESCC**

|  | β | SE | Walds | P | OR | 95% CI | Tolerance | VIF |
| --- | --- | --- | --- | --- | --- | --- | --- | --- |
| HB (g/L)  NEU (10^9^/L)  PLT (10^9^/L)  MON (10^9^/L)  LYM (10^9^/L)  ALB (g/dL)  BMI (Kg/m^2^)  PNI  NLR | 0.018  -0.159  -0.001  -1.184  0.687  0.866  0.140  0.083  -0.359 | 0.010  0.096  0.002  1.034  0.348  0.392  0.075  0.032  0.127 | 3.059  2.722  0.070  1.312  3.895  4.872  3.513  6.774  7.967 | 0.080  0.099  0.791  0.252  0.048  0.027  0.061  0.009  0.005 | 1.018  0.853  0.999  0.306  1.988  2.378  1.151  1.086  0.698 | 0.998-1.040  0.706-1.030  0.995-1.004  0.040-2.321  1.005-3.932  1.102-5.131  0.994-1.333  1.021-1.156  0.544-0.896 | 0.992  0.845  0.956  0.567  0.772  0.923  0.999  0.821  0.633 | 1.008  1.183  1.046  1.763  1.295  1.084  1.001  1.218  1.579 |
| PLR  LMR  SII  SIRI  HALP | -0.006  0.350  -0.001  -0.398  0.022 | 0.003  0.110  0.000  0.177  0.009 | 4.580  10.041  4.056  5.038  5.874 | 0.032  0.002  0.044  0.025  0.015 | 0.994  1.419  0.999  0.672  1.022 | 0.989-1.000  1.143-1.762  0.999-1.000  0.474-0.951  1.004-1.040 | 0.758  0.999  0.717  0.553  0.752 | 1.320  1.001  1.394  1.807  1.329 |

**Abbreviation:** ESCC: esophageal squamous cell carcinoma; SE: standard error; HB: hemoglobin; NEU: neutrophil; PLT: platelet; MON: monocyte; LYM: lymphocyte; ALB: albumin; BMI: body mass index; PNI: prognostic nutritional index; NLR: neutrophil to lymphocyte ratio; PLR: platelet to lymphocyte ratio; SII: systemic immune-inflammation index; LMR: lymphocyte to monocyte ratio; SIRI: systemic inflammation response index; HALP: hemoglobin albumin lymphocyte platelet; OR: odds ratio; CI: confidence interval; VIF: variance inflation factor.

**Table S3 Multivariate logistic analyses for pCR prediction based on various indices in ESCC**

|  | β | SE | Walds | P-value | OR | 95% CI |
| --- | --- | --- | --- | --- | --- | --- |
| BMI (Kg/m^2^)  LMR  Constant | 0.157  0.364  -5.657 | 0.077  0.112  1.786 | 4.211  10.633  10.029 | 0.040  0.001  0.002 | 1.171  1.440 | 1.007-1.361  1.156-1.792 |

**Abbreviation:** ESCC: esophageal squamous cell carcinoma; SE: standard error; BMI: body mass index; LMR: lymphocyte to monocyte ratio; OR: odds ratio; CI: confidence interval.

**Table S4 Comparative analysis of the AUC discrimination between BLS and other indices in ESCC**

| Index | AUC | AUC difference | Z-statistics | P-value |
| --- | --- | --- | --- | --- |
| BLS  HB  NEU  MON  PLT  ALB  BMI  NLR  PLR  LMR  PNI  SII  SIRI  HALP  BMI  PLR  SII  SIRI  LMR  PLR  SII  SIRI | 0.672 (0.603-0.735)  0.558 (0.487-0.627)  0.560 (0.489-0.629)  0.557 (0.486-0.626)  0.503 (0.432-0.573)  0.573 (0.502-0.641)  0.623 (0.553-0.690)  0.616 (0.546-0.683)  0.574 (0.503-0.642)  0.642 (0.572-0.707)  0.601 (0.531-0.669)  0.578 (0.508-0.647)  0.613 (0.543-0.680)  0.607 (0.536-0.674)  0.623 (0.553-0.690)  0.574 (0.503-0.642)  0.578 (0.508-0.647)  0.613 (0.543-0.680)  0.642 (0.572-0.707)  0.574 (0.503-0.642)  0.578 (0.508-0.647)  0.613 (0.543-0.680) | reference  0.114 (0.0055-0.222)  0.112 (0.0268-0.197)  0.115 (0.0460-0.184)  0.169 (0.0382-0.300)  0.099 (0.0047-0.193)  0.049 (-0.0400-0.137)  0.056 (-0.0164-0.127)  0.098 (0.0143-0.182)  0.030 (-0.0162-0.076)  0.071 (-0.0124-0.154)  0.094 (0.0169-0.170)  0.059 (0.0023-0.116)  0.065 (-0.0171-0.147)  reference  0.049 (-0.0680-0.168)  0.045 (-0.0773-0.167)  0.010 (-0.1140-0.135)  reference  0.068 (-0.0148-0.152)  0.064 (-0.0077-0.135)  0.029 (-0.0078-0.066) | reference  2.059  2.575  3.267  2.531  2.057  1.073  1.513  2.294  1.270  1.667  2.394  2.040  1.551  reference  0.829  0.721  0.168  reference  1.612  1.747  1.548 | reference  0.0395  0.0100  0.0011  0.0114  0.0397  0.2832  0.1303  0.0218  0.2040  0.0954  0.0167  0.0413  0.1208  reference  0.4072  0.4712  0.8667  reference  0.1070  0.0806  0.1216 |

**Abbreviation:** ESCC: esophageal squamous cell carcinoma; AUC: area under the curve; HB: hemoglobin; NEU: neutrophil; PLT: platelet; MON: monocyte; LYM: lymphocyte; ALB: albumin; BMI: body mass index; PNI: prognostic nutritional index; NLR: neutrophil to lymphocyte ratio; PLR: platelet to lymphocyte ratio; SII: systemic immune-inflammation index; SIRI: systemic inflammation response index; LMR: lymphocyte to monocyte ratio; HALP: hemoglobin albumin lymphocyte platelet.

**Table S5** **Logistic analyses for clinical characteristics with pCR in ESCC**

|  | Univariate analysis  OR (95% CI) P-value | Multivariate analysis  OR (95% CI) P-value |
| --- | --- | --- |
| Sex (male vs. female)  Age (years, >70 vs. ≤70)  Hypertension (yes vs. no)  Diabetes (yes vs. no)  Smoking history (yes vs. no)  Drinking history (yes vs. no)  Tumor location  upper  middle  lower  Differentiation  well  moderate  poor  cTNM stage  II  III  IVa  Immunotherapy  camrelizumab  pembrolizumab  nivolumab  sintilimab  tislelizumab | 0.679 (0.235-1.959) 0.474  0.663 (0.294-1.497) 0.323  0.602 (0.297-1.220) 0.159  0.581 (0.120-2.816) 0.500  0.566 (0.299-1.072) 0.081  0.676 (0.355-1.289) 0.235  0.777  reference  1.373 (0.465-4.055) 0.566  1.149 (0.364-3.624) 0.813  0.013  reference  0.548 (0.242-1.245) 0.151  0.275 (0.114-0.665) 0.004  0.007  reference  0.432 (0.203-0.922) 0.030  0.146 (0.042-0.504) 0.002  0.332  reference  0.628 (0.246-1.601) 0.330  0.439 (0.090-2.132) 0.307  1.480 (0.479-4.574) 0.496  0.509 (0.213-1.215) 0.128 | 0.029  reference  0.606 (0.248-1.477) 0.270  0.289 (0.110-0.756) 0.011  0.040  reference  0.580 (0.254-1.322) 0.195  0.184 (0.050-0.679) 0.011 |
| BLS (low vs. high) | 0.285 (0.151-0.538) <0.001 | 0.269 (0.138-0.527) <0.001 |

**Abbreviation:** ESCC: esophageal squamous cell carcinoma; pCR: pathological complete response; BLS: BMI-LMR score; OR: odds ratio; CI: confidence interval; TNM: tumor node metastasis.

**Table S6 Cox analyses of prognostic factors associated with DFS in ESCC**

|  | Univariate analysis  HR (95% CI) P-value | Multivariate analysis  HR (95% CI) P-value |
| --- | --- | --- |
| Sex (male vs. female)  Age (years, >70 vs. ≤70)  Hypertension (yes vs. no)  Diabetes history (yes vs. no)  Smoking history (yes vs. no)  Drinking history (yes vs. no)  Tumor location  upper  middle  lower  Differentiation  well  moderate  poor  Vessel invasion (yes vs. no)  Perineural invasion (yes vs. no)  Tumor length (cm, >3 vs. ≤3)  ypT stage  T0  T1-2  T3-4a  ypN stage (N1-3 vs. N0)  Immunotherapy  camrelizumab  pembrolizumab  nivolumab  sintilimab  tislelizumab  Adjuvant treatment  none  immunotherapy  chemoradiotherapy  pCR (yes vs. no) | 1.025 (0.370-2.838) 0.962  0.467 (0.200-1.090) 0.078  0.680 (0.358-1.289) 0.237  1.055 (0.329-3.378) 0.928  0.890 (0.502-1.579) 0.691  1.373 (0.737-2.557) 0.318  0.002  reference  0.281 (0.137-0.574) 0.001  0.467 (0.224-0.978) 0.043  0.823  reference  1.281 (0.554-2.961) 0.563  1.293 (0.555-3.014) 0.551  3.780 (2.051-6.968) <0.001  1.716 (0.921-3.197) 0.089  2.306 (1.321-4.027) 0.003  0.007  reference  2.905 (1.228-6.871) 0.015  3.778 (1.650-8.653) 0.002  3.291 (1.908-5.679) <0.001  0.660  reference  1.179 (0.561-2.477) 0.663  0.845 (0.258-2.766) 0.781  0.238 (0.033-0.747) 0.158  0.930 (0.468-0.852) 0.837  0.193  reference  0.651 (0.356-1.190) 0.163  0.482 (0.172-1.355) 0.166  0.165 (0.060-0.457) 0.001 | 0.007  reference  0.313 (0.150-0.653) 0.002  0.382 (0.180-0.809) 0.012  2.347 (1.234-4.462) 0.009  2.632 (1.477-4.689) 0.001 |
| BLS (low vs. high) | 2.494 (1.393-4.466) 0.002 | 2.570 (1.427-4.628) 0.002 |

**Abbreviation:** ESCC: esophageal squamous cell carcinoma; pCR: pathological complete response; BLS: BMI-LMR score; HR: hazard ratio; CI: confidence interval; DFS: disease-free survival.

**Table S7 Cox analyses of prognostic factors associated with OS in ESCC**

|  | Univariate analysis  HR (95% CI) P-value | Multivariate analysis  HR (95% CI) P-value |
| --- | --- | --- |
| Sex (male vs. female)  Age (years, >70 vs. ≤70)  Hypertension (yes vs. no)  Diabetes history (yes vs. no)  Smoking history (yes vs. no)  Drinking history (yes vs. no)  Tumor location  upper  middle  lower  Differentiation  well  moderate  poor  Vessel invasion (yes vs. no)  Perineural invasion (yes vs. no)  Tumor length (cm, >3 vs. ≤3)  ypT stage  T0  T1-2  T3-4a  ypN stage (N1-3 vs. N0)  Immunotherapy  camrelizumab  pembrolizumab  nivolumab  sintilimab  tislelizumab  Adjuvant treatment  none  immunotherapy  chemoradiotherapy  pCR (yes vs. no) | 0.616 (0.187-2.033) 0.427  0.417 (0.127-1.372) 0.150  0.621 (0.254-1.516) 0.295  0.616 (0.084-4.517) 0.633  0.848 (0.389-1.849) 0.678  1.642 (0.673-4.004) 0.276  0.076  reference  0.329 (0.126-0.860) 0.023  0.451 (0.166-1.225) 0.118  0.435  reference  2.054 (0.602-7.010) 0.251  1.477 (0.412-5.298) 0.549  2.494 (1.013-6.143) 0.047  2.113 (0.972-4.592) 0.059  2.115 (0.992-4.511) 0.053  0.073  reference  2.620 (0.834-8.233) 0.099  3.592 (1.200-10.753) 0.022  4.615 (2.123-10.033) <0.001  0.717  reference  1.809 (0.406-2.918) 0.866  0.789 (0.182-3.408) 0.750  0.398 (0.053-2.971) 0.369  0.543 (0.184-1.603) 0.269  0.488  reference  0.623 (0.274-1.416) 0.258  0.687 (0.204-2.312) 0.544  0.158 (0.038-0.662) 0.012 | 0.038  reference  0.338 (0.127-0.897) 0.029  0.275 (0.098-0.771) 0.014  4.538 (2.022-10.182) <0.001 |
| BLS (low vs. high) | 3.904 (1.600-9.522) 0.003 | 3.827 (1.559-9.396) 0.003 |

**Abbreviation:** ESCC: esophageal squamous cell carcinoma; pCR: pathological complete response; BLS: BMI-LMR score; HR: hazard ratio; CI: confidence interval; OS: overall survival.
